# Supplementary material for: Comparative transcriptomics of leaves of five mulberry accessions and cataloguing structural and expression variants for future prospects
Source: PLoS One. 2021 Jul 14;16(7):e0252246. doi: 10.1371/journal.pone.0252246 (PMC8279327; doi:10.1371/journal.pone.0252246)

Supplementary figure 1 showing the network of cellular component enriched GO terms for the unique genes of K2 (MI) genotype. Nodes represent the GO terms. Size of the nodes represents the number of gene and the colour represent significant value (See scale).

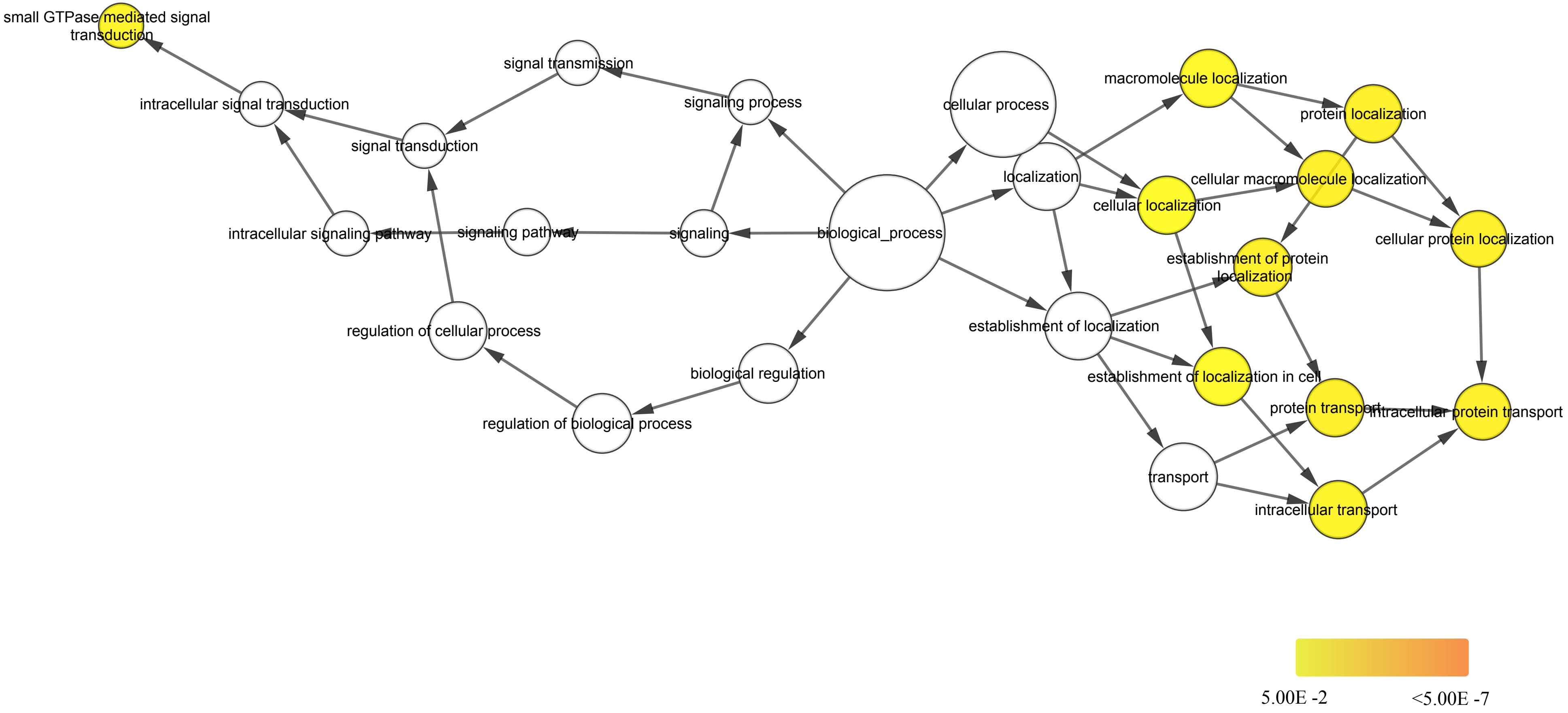

Supplementary figure 2 showing the network of molecular function enriched GO terms for the unique genes of K2 (MI) genotype. Nodes represent the GO terms. Size of the nodes represents the number of gene and the colour represent significant value (See scale).

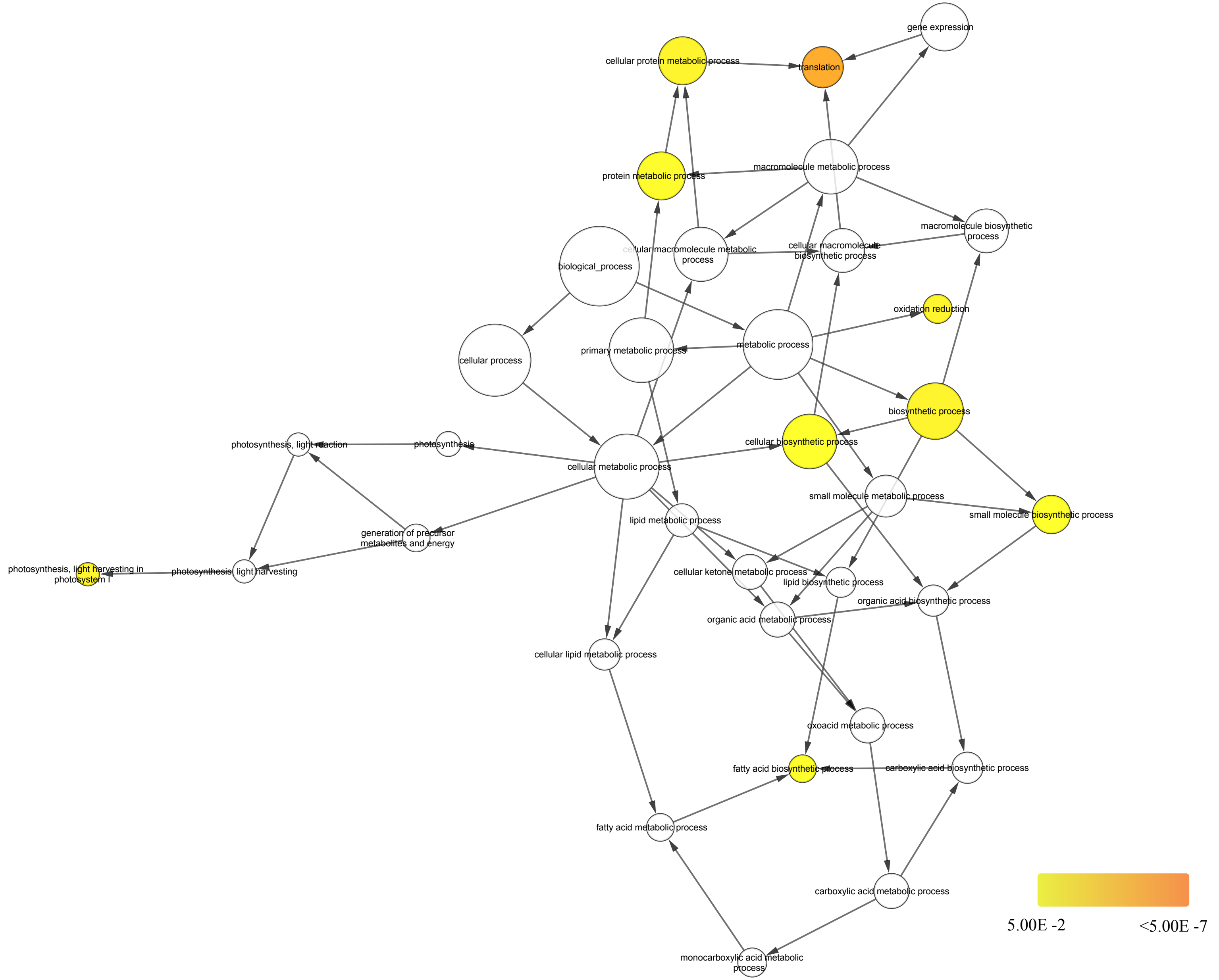

Supplementary figure 3 showing the network of biological process enriched GO terms for the unique genes of ML genotype. Nodes represent the GO terms. Size of the nodes represents the number of gene and the colour represent significant value (See scale).

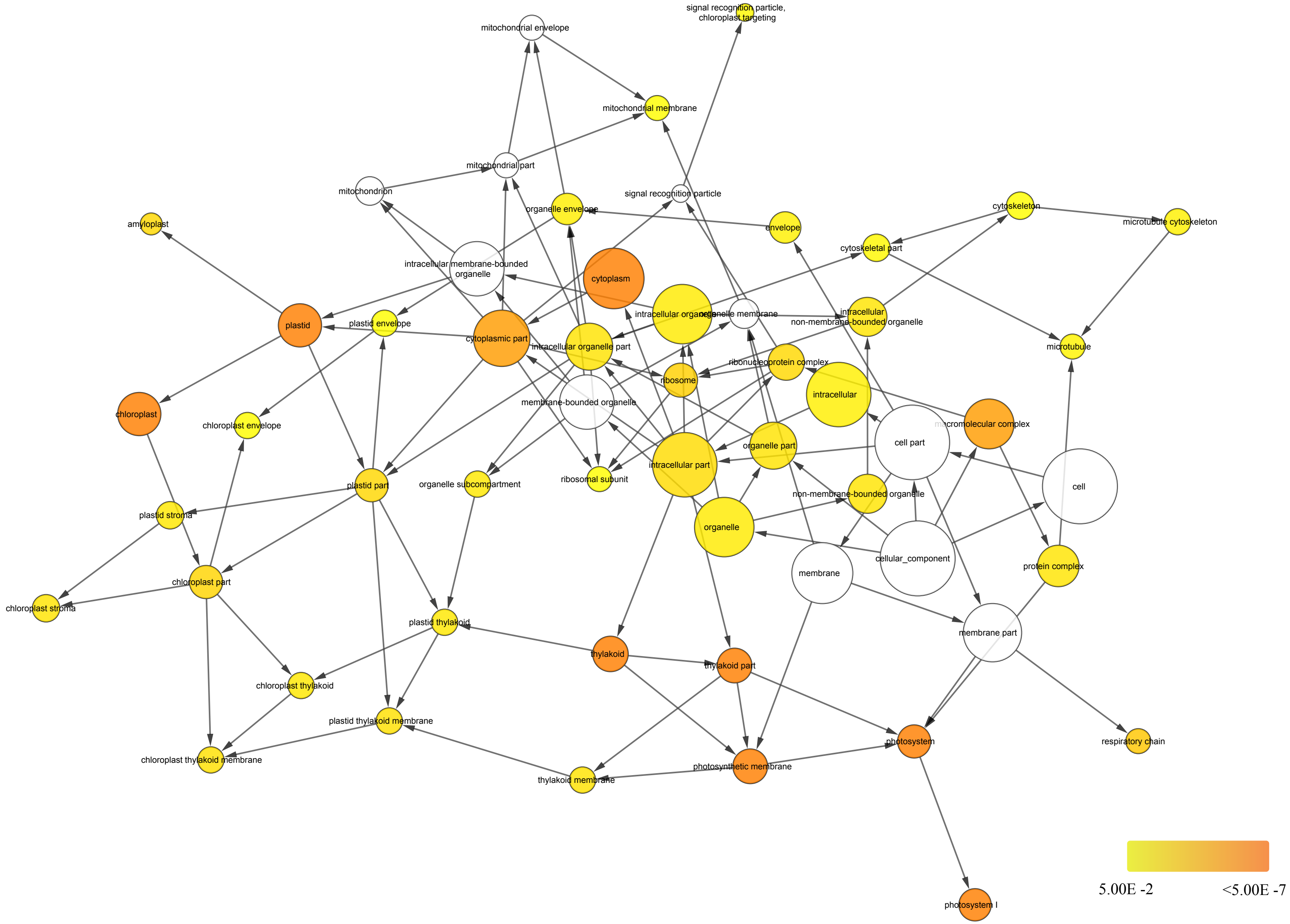

Supplementary figure 4 showing the network of cellular component enriched GO terms for the unique genes of ML genotype. Nodes represent the GO terms. Size of the nodes represents the number of gene and the colour represent significant value (See scale).

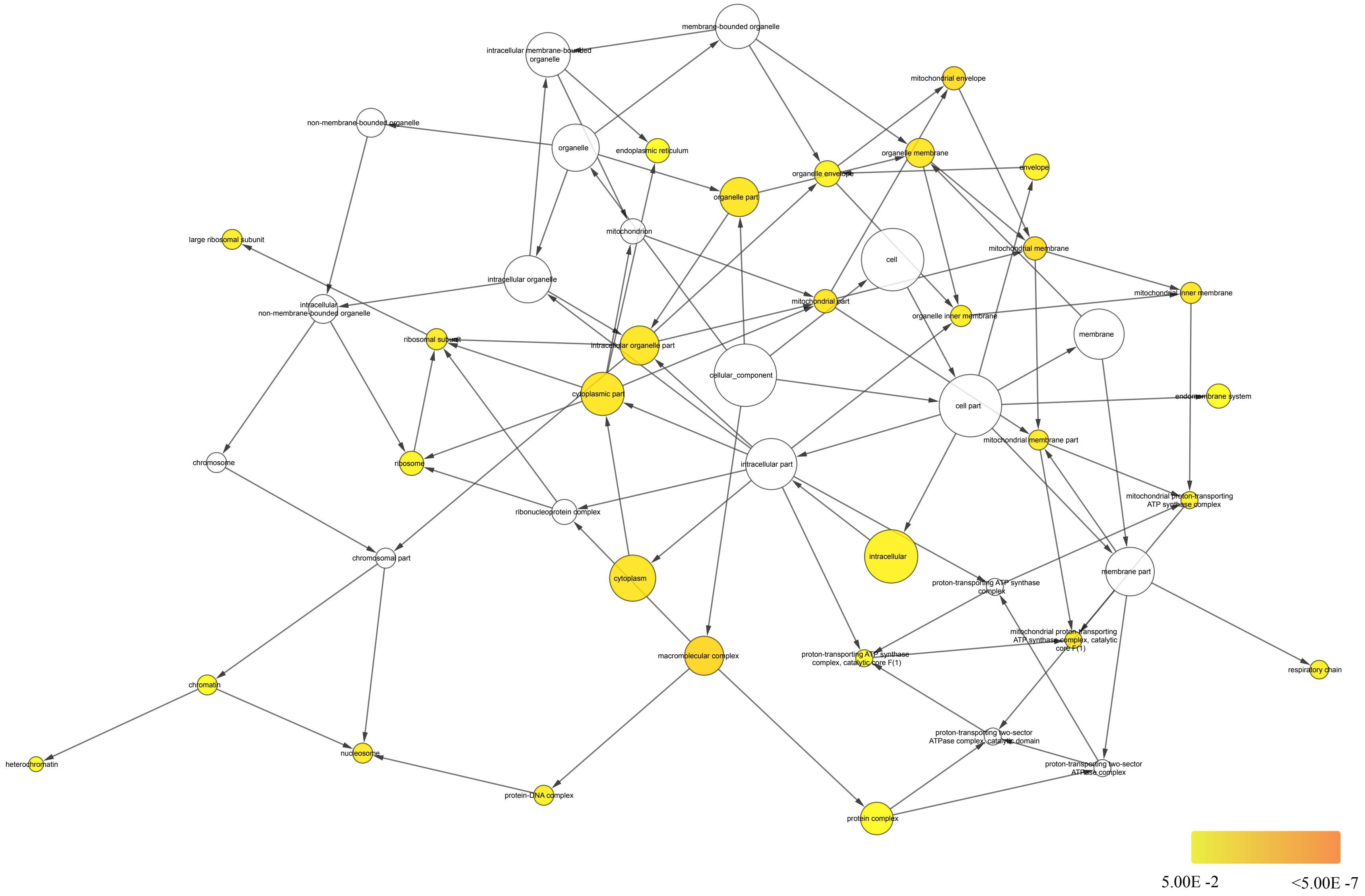

Supplementary figure 5 showing the network of molecular function enriched GO terms for the unique genes of ML genotype. Nodes represent the GO terms. Size of the nodes represents the number of gene and the colour represent significant value (See scale).

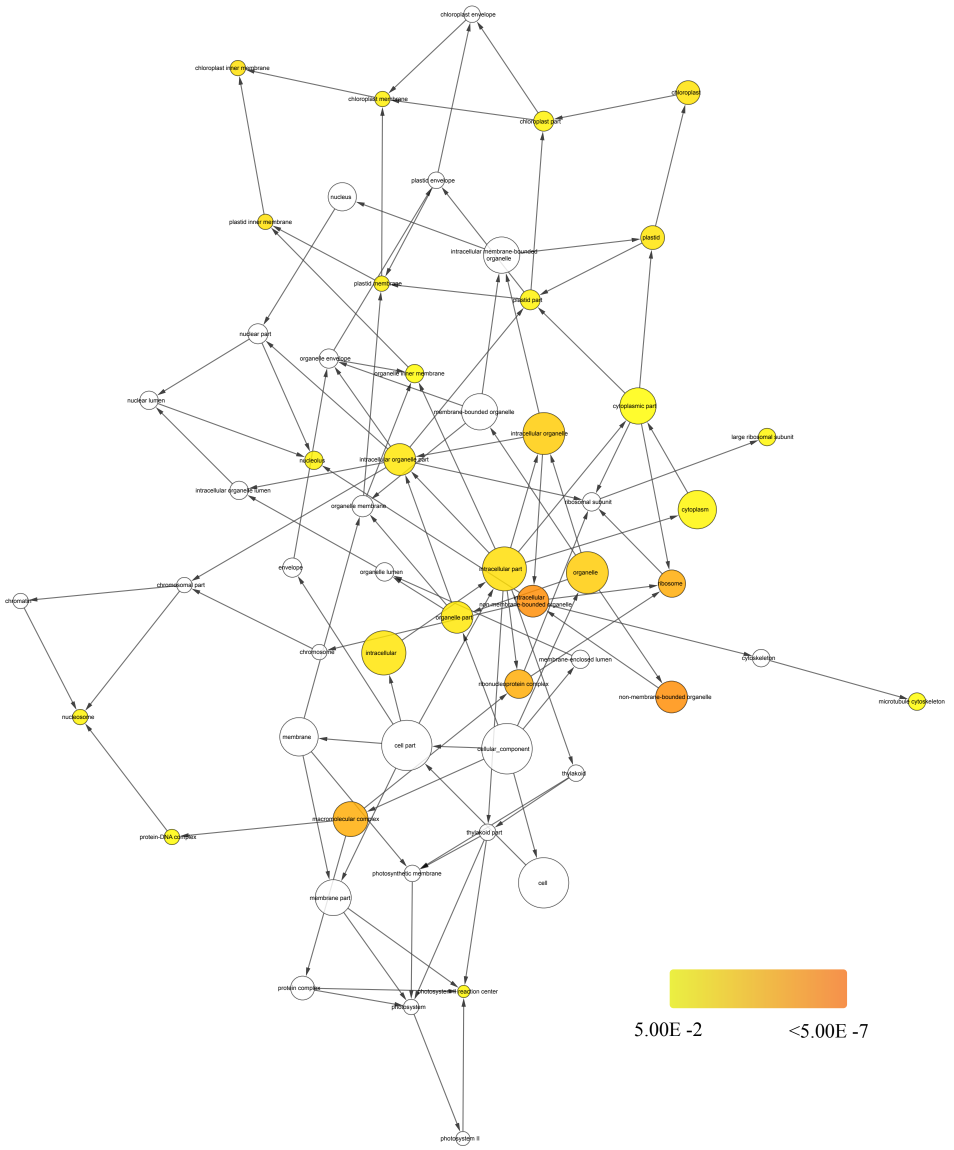

Supplementary figure 6 showing the network of cellular component enriched GO terms for the unique genes of MN genotype. Nodes represent the GO terms. Size of the nodes represents the number of gene and the colour represent significant value (See scale).

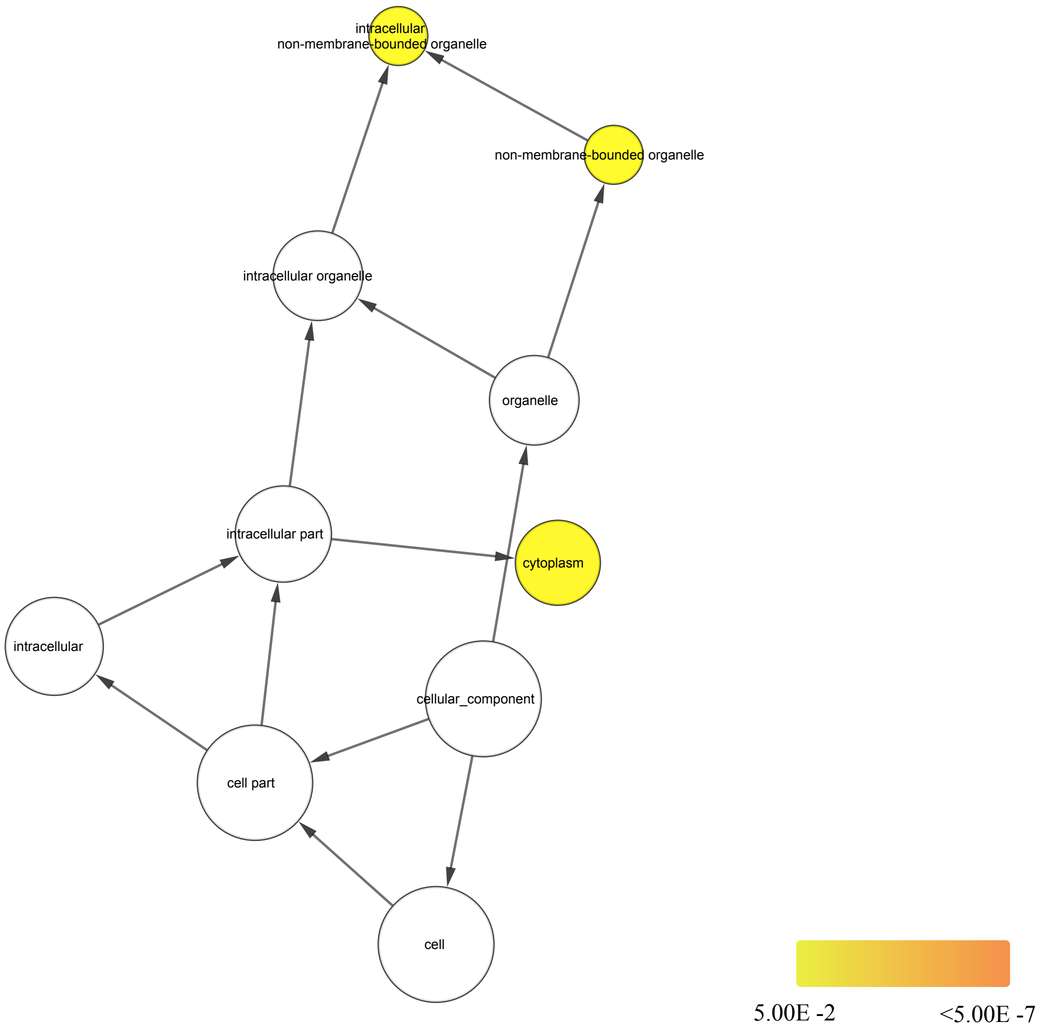

Supplementary figure 7 showing the network of molecular function enric hed GO terms for the unique genes of MN genotype. Nodes represent the GO terms. Size of the nodes represents the number of gene and the colour represent significant value (See scale).

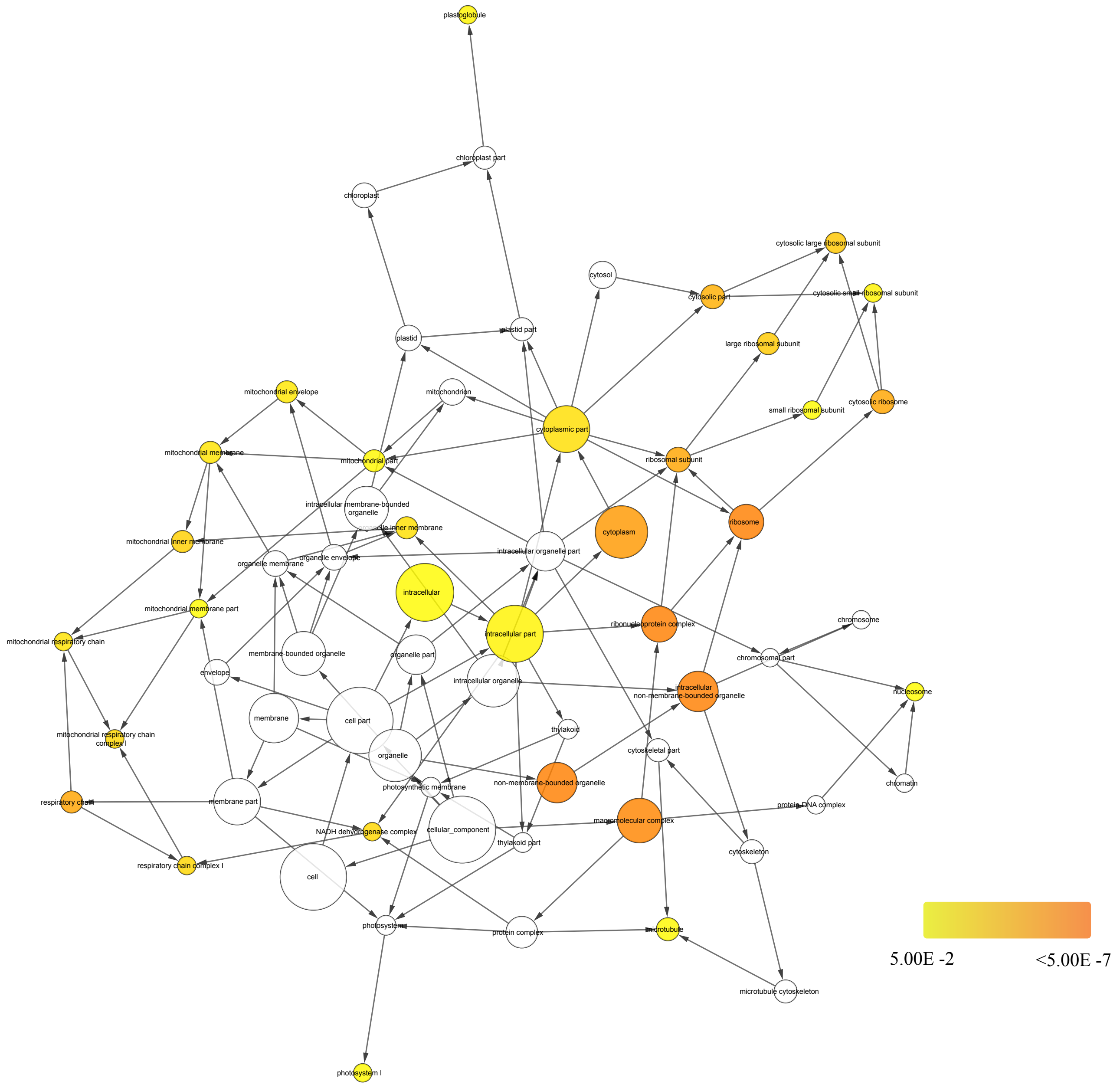

Supplementary figure 8 showing the network of cellular component enriched GO terms for the unique genes of MS genotype. Nodes represent the GO terms. Size of the nodes represents the number of gene and the colour represent significant value (See scale).

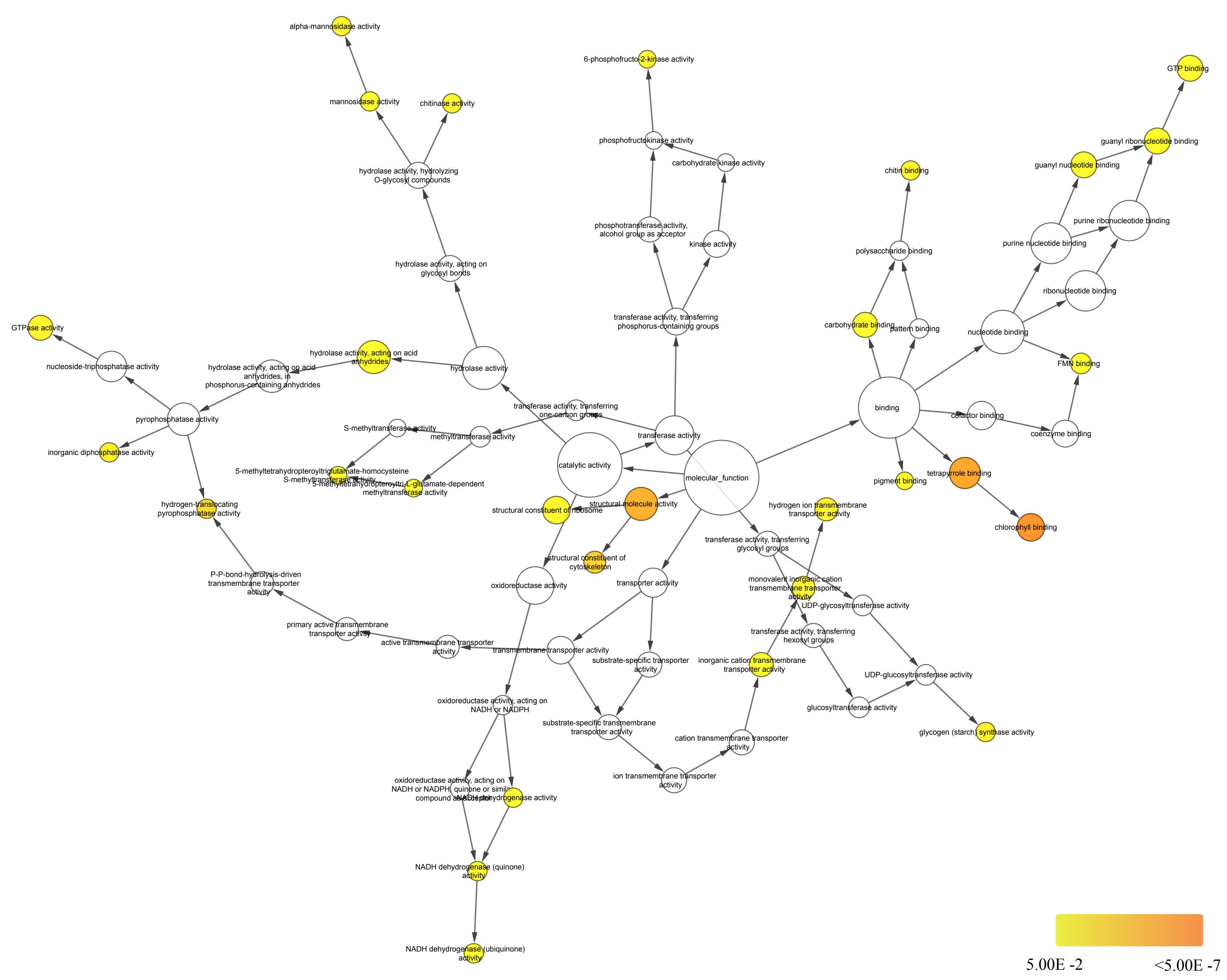

Supplementary figure 9 showing the network of biological process enriched GO terms for the unique genes of V (MI) genotype. Nodes represent the GO terms. Size of the nodes represents the number of gene and the colour represent significant value (See scale).

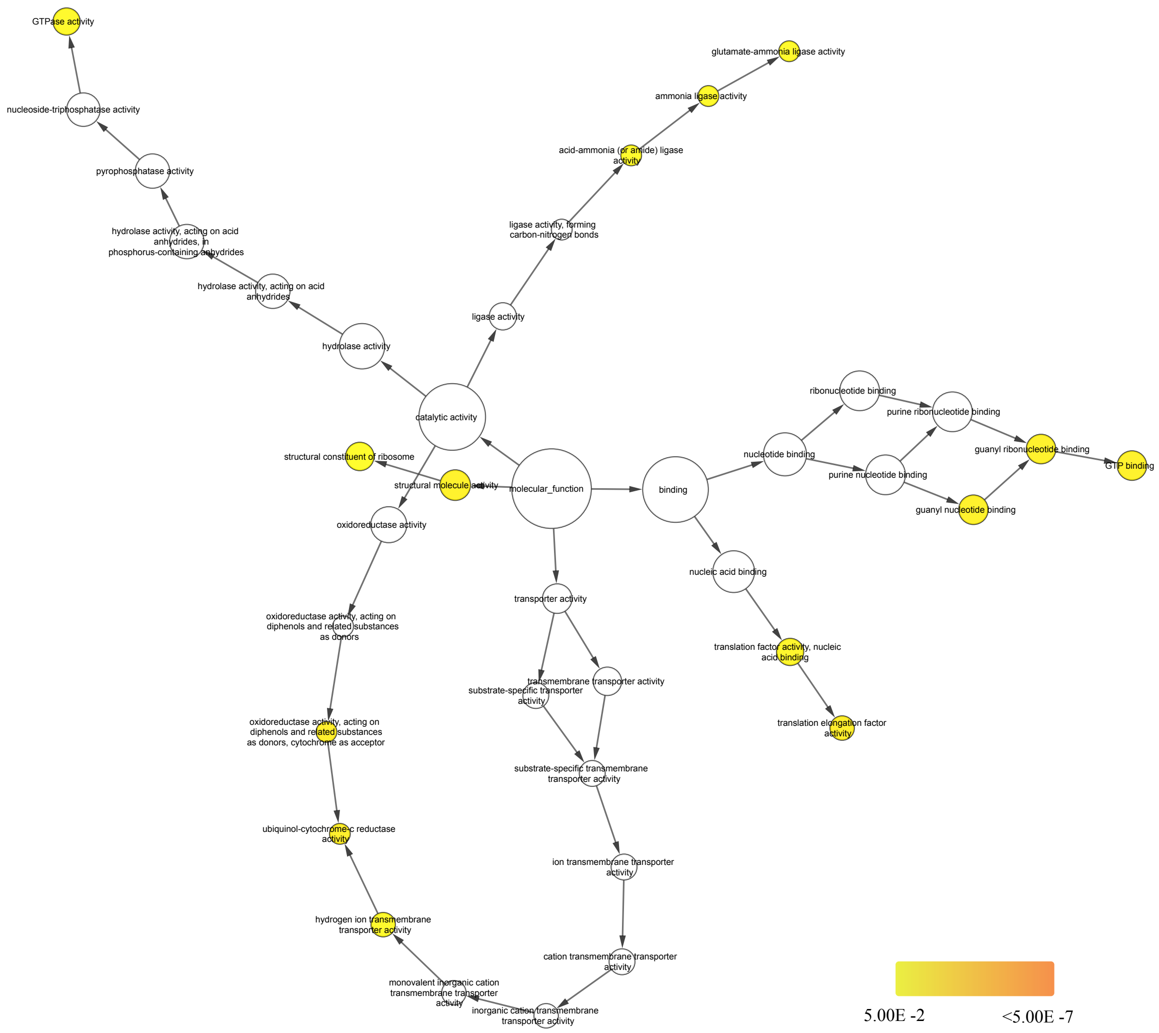

Supplementary figure 10 showing the network of cellular component enriched GO terms for the unique genes of V (MI) genotype. Nodes represent the GO terms. Size of the nodes represents the number of gene and the colour represent significant value (See scale).

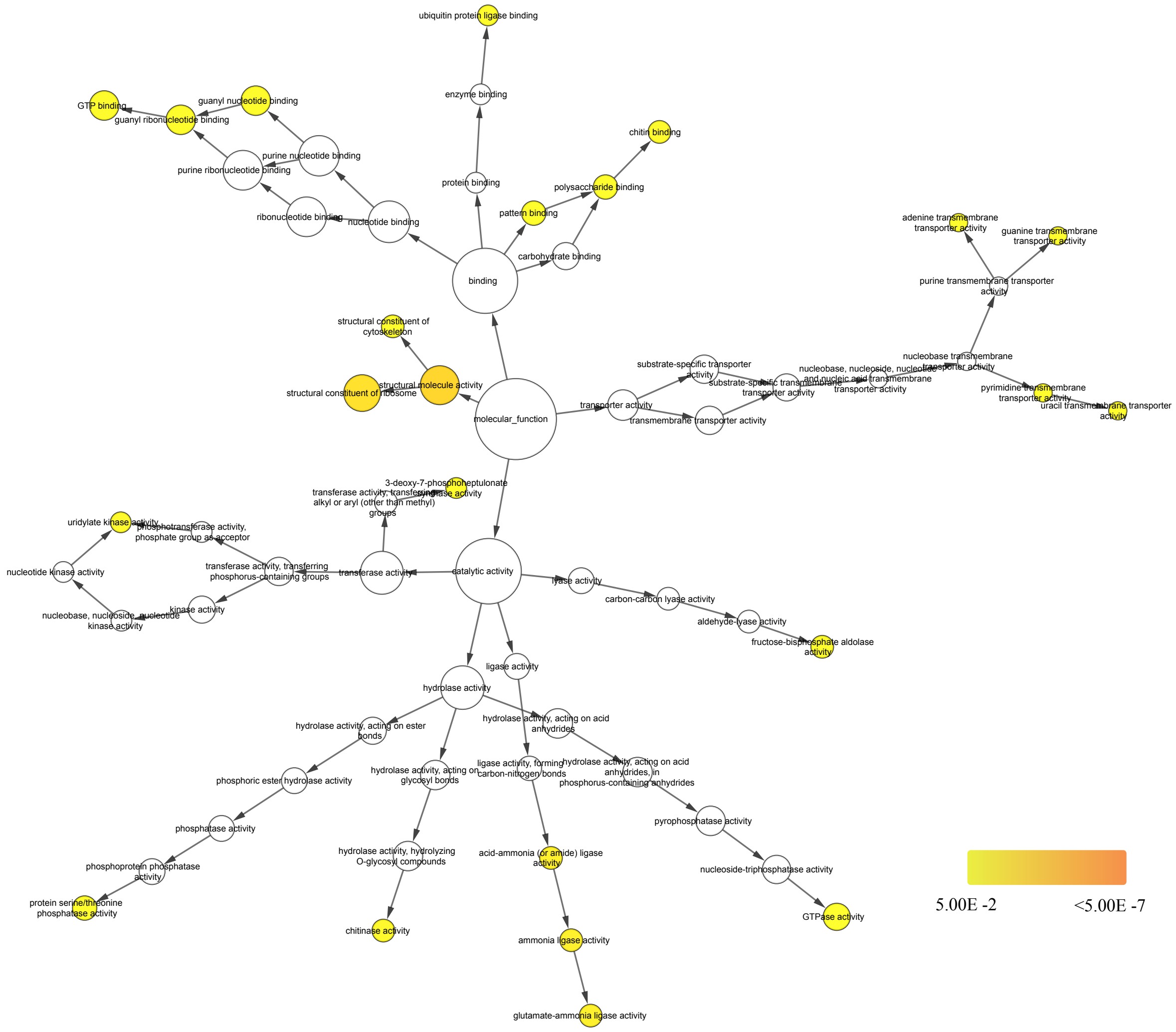

Supplementary figure 11 showing the network of molecular function enriched GO terms for the unique genes of V (MI) genotype. Nodes represent the GO terms. Size of the nodes represents the number of gene and the colour represent significant value (See scale).

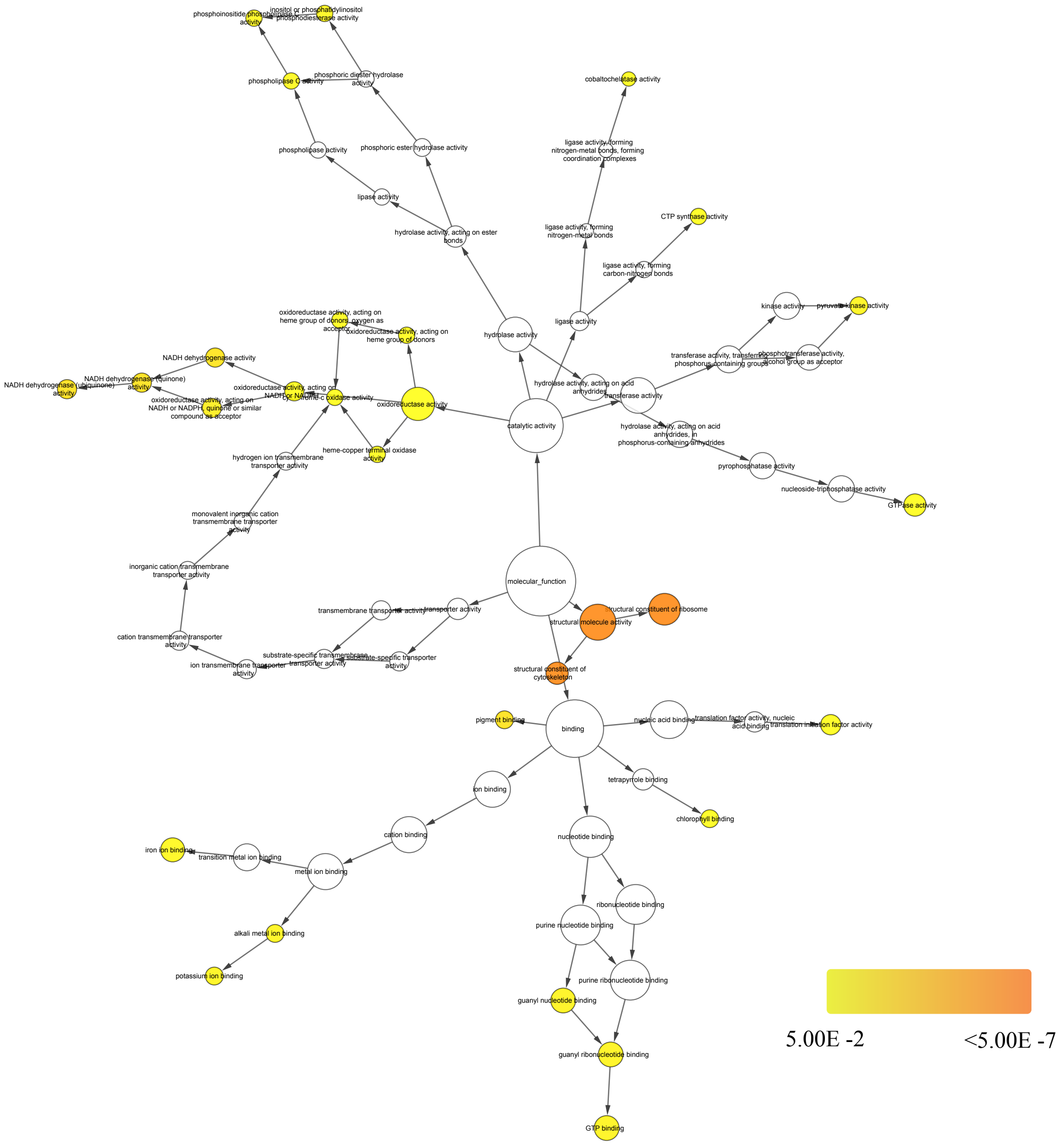

Supplement: S1 File — (PDF) [file pone.0252246.s004.pdf]
